# Pathologists' user experience in the era of digital pathology: a KAP study in a region of emerging digitization

**DOI:** 10.3389/fdgth.2025.1603985

**Published:** 2025-09-01

**Authors:** Maher A. Sughayer, Lina Souan, Joud S. Tadros

**Affiliations:** ^1^ Department of Pathology and Laboratory Medicine, King Hussein Cancer Center, Amman, Jordan; ^2^ School of Medicine, Jordan University, Amman, Jordan

**Keywords:** Jordan, digital pathology, artificial intelligence, survey, perception

## Abstract

**Background:**

Information regarding the use of digital pathology (DP) in developing countries is limited. Additionally, the knowledge and attitudes/perceptions of pathologists are mainly unknown. In this study, we aim to assess the knowledge and attitudes of Jordanian pathologists on DP and artificial intelligence (AI).

**Methods:**

A digital survey consisting of 32 questions was constructed using Google Forms and sent to practicing pathologists across all sectors in Jordan. The results were analyzed using descriptive statistics.

**Results:**

Forty pathologists representing university hospitals, the Ministry of Health, the Royal Medical Services (RMS), and the private sector (PS) participated in the study. 69.2% of participants had average/above-average knowledge of DP. 77.8% of participants without scanners were interested in obtaining one if funds were available, and 85% were likely or very likely to use it for diagnostic purposes. In comparison, 92.5% were very likely to use it for consultation. Cases diagnosed using DP represent 10%. 85% of participants expressed interest in attending sessions at a national congress on DP, and 37.5% currently use AI platforms. Approximately 65% of people with DP didn't follow any guidelines. Seventy-one percent and twenty-nine percent of the guidelines used were from the College of American Pathologists (CAP) and the Royal College of Pathologists (RCP), respectively. At the same time, all pathologists believed the Jordanian Pathologists Society should develop its guidelines. 76.9% thought that a lack of funds was the primary obstacle to adopting DP. In comparison, a lack of infrastructure and experience ranked second, with 40% indicating a lack of interest or a preference for glass slides as obstacles. As for the primary use of DP, 86.8%, 73.7%, 63.2%, 50%, 44.7%, and 44.7% would use it for consultation, education, research, diagnosis, archiving cases, and tumor boards, respectively.

**Conclusions:**

Although digital pathology and slide scanners are limited in Jordan, most pathologists are willing to adopt their use, provided that the significant challenges of a lack of funding and inadequate infrastructure are addressed. The primary uses of DP in Jordan seem to be related to consultations and research.

## Introduction

The medical field has experienced significant technological advancements since the turn of the 21st century. This includes transitioning from radiographic film to radiological digital imaging and adopting minimally invasive laparoscopies instead of invasive open operations (1). These advancements have revolutionized the medical field in nearly all areas, resulting in improved patient outcomes and extended life expectancies (2). Although the medical world has witnessed significant advancements, anatomical pathology has remained a relatively quiet analog specialty, with pathologists preferring traditional glass slide preparation, staining, and bright-field microscopy techniques (3). This is primarily due to the lack of aspects of digital sensors and imaging techniques that do not yet meet the standards of good medical practice.

In recent years, the notion of digital pathology (DP) has evolved, transforming the profession by mixing contemporary technologies and advanced tools with pathology. Digital pathology has facilitated the advancement of slide scanner technologies and processing capabilities. The display and storage of slides have been enhanced, becoming more accessible and readily available (4). It enables substantial archiving of slides, hence facilitating seamless access and data sharing with other universities. This simplifies collaborative care networks and integration among national and international organizations (5).

Additionally, it represents a novel approach that converts conventional glass slides into high-resolution digital images, which can be viewed, analyzed, stored, and shared online. This process relies on Whole Slide Imaging (WSI), which enables the scanning of glass slides and the conversion of these images into digital formats that can be viewed on a monitor (6, 7). DP is significantly enhanced by its integration with artificial intelligence (AI), which facilitates the detection and grading of tumors (8–10).

The advantages of DP are well-documented and widely recognized. It increases diagnostic accuracy and efficiency, enhances workflow productivity, and reduces human error. It eases remote diagnosis and promotes educational and research opportunities (11). It is worth noting that DP has several disadvantages. Some of these disadvantages include its high cost, technical challenges, regulatory uncertainties, and the need for new training, which pose the main obstacles to adopting such a method, especially in low- to middle-income countries with less established healthcare systems (12, 13).

Pathologists' perceptions of DP vary widely globally. Pathologists in well-established healthcare infrastructures, such as the United States and Europe, are optimistic about this technology, recognizing its potential to revolutionize routine diagnostics, medical education, and research (14). On the other hand, pathologists in the Middle East are enthusiastic about modernization but hesitant due to the region's unique challenges. For example, studies conducted in Saudi Arabia and Jordan reveal that while pathologists recognize the importance of adopting DP and AI in their practice, they face various obstacles, including limited financial resources, insufficient technical infrastructure, and a lack of standardized protocols for implementation (15–18). The literature review reveals that pathologists' perceptions of DP have not yet been fully explored, and they continue to hold mixed opinions about it. Hence, in this study, we aimed to investigate the perceptions and attitudes of Jordanian pathologists toward digital pathology and AI, providing insights into the benefits, obstacles, and factors influencing its adoption into practice.

## Materials and methods

### Survey setting and design

This online survey study was conducted at the King Hussein Cancer Center (KHCC) from August 2 to August 9, 2024. Google Forms (Google LLC). The survey used in this study was developed electronically based on the study objectives and relevant literature. To ensure the clarity, relevance, and appropriateness of the content, the survey underwent face and content validation through expert review by the project's Principal Investigator (PI), who also conducted a pilot test with a small group representative of the target population. Feedback from this pilot testing was used to refine the wording and structure of the questions, thereby enhancing the survey's validity and usability before full deployment. The survey was written in English and was sent via WhatsApp, a social media application, to the registered pathologists in the Jordanian Board of Pathologists. The mailing list included the nationalities and institutions of the pathologists' workplaces. Pathologists working in the private sector, public sector, and universities were equally included in the mailing list.

### Sample size estimation and sampling strategy

The required sample size was determined using power analysis, ensuring adequate representation of the study population. Based on prior similar studies and considering 100 Jordanian pathologists registered with the medical association, a sample of at least 40 pathologists was expected to provide reliable statistical insights, assuming a margin of error of ±12.07% at a 95% confidence level, calculated using the standard finite population correction formula for proportions.

The survey included several sections, and various types of answer questions were used, including short open-ended questions, multiple-choice questions, checkbox questions, and Likert scales (ranging from 1 to 5). The participants were asked to give basic information about themselves, their institution, and whether they have available digital slide scanners in their laboratories.

On the one hand, if the respondents indicated that digital slide scanners were available, they were asked about the number and type of scanners, the frequency of use, and the primary reasons for using them. They were also asked about the guidelines they follow and their opinions on these guidelines. On the other hand, if the question of the availability of digital slide scanners was no, the respondents were asked about their interest in obtaining one for their institution and the challenges that prevented the adoption of these scanners in their institutions.

All respondents were then asked for their opinions on the advantages and challenges of adopting digital pathology in institutions and its impact on the workflow of laboratories in Jordan. Specific challenges were highlighted, including a lack of funding, slow internet speeds, insufficient storage, and a shortage of skills. Opinions on devising and following national regulatory guidelines shall be collected. Respondents were also asked about the availability of laboratory information systems and barcoding in their laboratories and their openness to using AI tools in the pathology lab. The complete list of survey questions is provided in the Supplementary Appendix section.

## Selection criteria and recruitment strategy

### Sampling strategy

A mixed-methods sampling strategy was employed, using stratified sampling to ensure a balanced representation across key subgroups (e.g., years of experience, region, type of practice). Convenience and Snowball Sampling were employed, and pathologists were recruited through professional associations and institutional contacts. Participants were encouraged to refer colleagues to increase response rates.

### Ethical clearance of the study

Participants were provided with detailed information regarding the study's purpose, procedures, confidentiality assurances, and their rights either via WhatsApp or by phone, and participation was entirely voluntary, with completion of the survey considered an indication of informed consent; this consent procedure was reviewed and approved by the Institutional Review Board of King Hussein Cancer Center, Amman, Jordan (IRB #25 KHCC 062) following ethical guidelines for online research involving human participants.

### Statistical analysis

The responses were collected in a Microsoft Excel spreadsheet (Microsoft) through Google Forms statistics. After the form closed, duplicate, incomplete, or incomprehensive responses were removed from the database, and the valid responses remaining were analyzed. Descriptive analysis was performed using Google Forms statistics.

## Results

### Geographical distribution of the institutions

**Figure 1:**
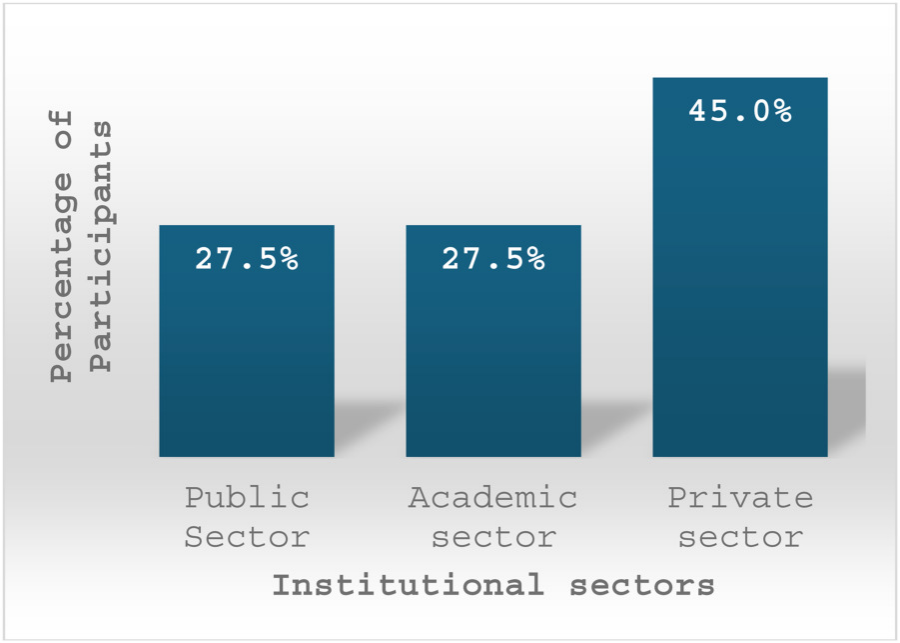
This bar chart illustrates the distribution of participants across different institutional affiliations.

We analyzed data across three institutional sectors: public, private, and academic. Participants were affiliated with various institutions, including public sectors such as Ministry of Health (MOH) [*n* = 6, (15%)], the Royal Medical Services [*n* = 5, (12.5%)], and Academic sectors such as universities [*n* = 11, (27.5%)]. The remaining participants were employed in private hospitals (*n* = 14, 35%). Additionally, four participants were from non-hospital-based private laboratories Figure 1.

### Participant demographics

A total of 40 participants were included in the study. The age distribution was as follows: 14 participants (35%) were under the age of 40, 18 participants (45%) were between 40 and 60 years old, and 8 participants (20%) were over 60 years old (Table 1). In terms of gender, the sample comprised 23 females (57.5%) and 17 males (42.5%) (Table 2).

**Table 1:** Distribution of participants by age group.

Age group	Count	Percentage (%)
Under 40	14	35.0
40–60	18	45.0
Over 60	8	20.0
Total	40	100.0

This table summarizes the distribution of study participants according to age group. Percentages are calculated based on the total sample size (*N* = 40).

**Table 2:** Distribution of participants by gender.

Gender	Count	Percentage (%)
Female (F)	23	57.5
Male (M)	17	42.5
Total	40	100.0

This table presents the gender distribution of participants included in the study. Percentages are based on the total number of respondents (*N* = 40).

### Knowledge of digital pathology and AI

A total of 69.2% of participants demonstrated an average or above-average level of knowledge in digital pathology, scoring three or higher on the knowledge scale. Regarding educational exposure, 50% of participants in academic institutions reported that their pathology curriculum included lectures on digital pathology and artificial intelligence (AI). Additionally, 45% of participants attended sessions related to AI, and 37.5% reported using AI platforms.

**Figure 2:**
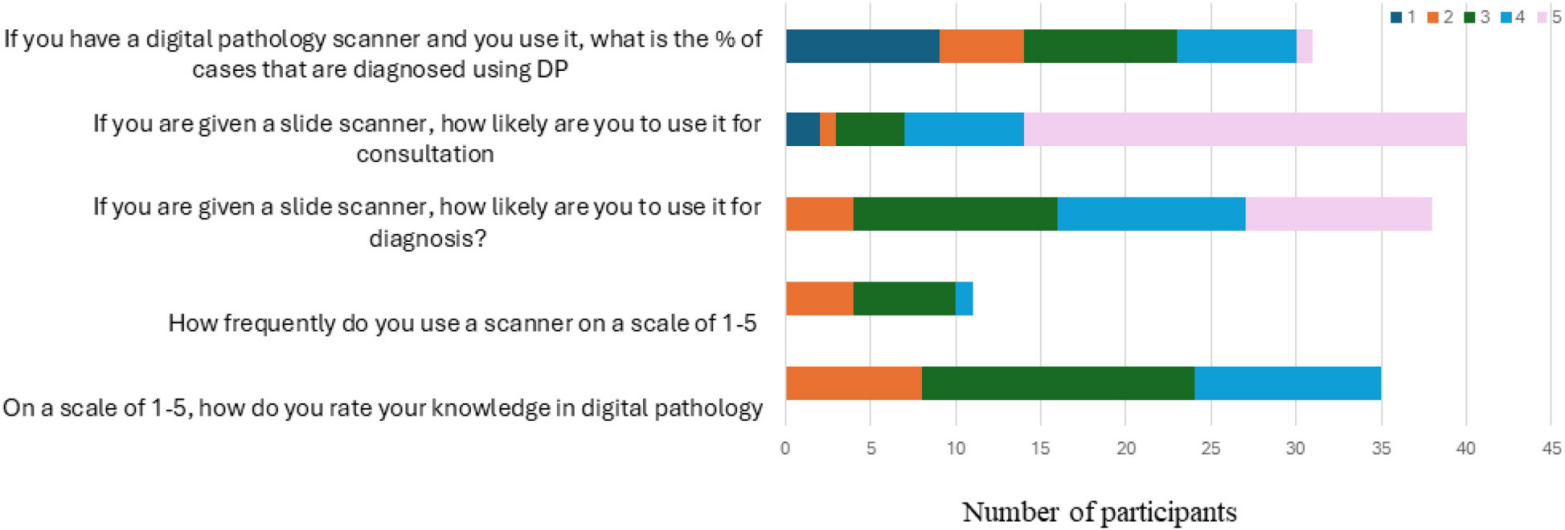
Knowledge and utilization of digital pathology.

Among AI users, 64.3% applied AI for research, while 46.4% used it for computer-aided diagnosis. This reflects the respondents' moderate familiarity and awareness of AI applications Figure 2.

Likert scale responses assessing participants' experience and attitudes toward digital pathology.

This figure presents participants' ratings on five survey items using a 5-point Likert scale. Items assessed included: (1) self-rated knowledge in digital pathology; (2) frequency of scanner use; (3) likelihood of using a slide scanner for diagnostic purposes; (4) likelihood of using a slide scanner for consultation; and (5) the estimated percentage of cases diagnosed using digital pathology when a scanner is available. Higher scores indicate greater knowledge, increased frequency of use, or a higher likelihood of adoption.

### Attitude toward digital pathology and AI

There was a high level of interest in advancing knowledge and integrating digital pathology into practice. 85% of participants expressed interest in attending a national congress session on digital pathology, and 77.8% of those without scanners indicated they would acquire a digital slide scanner if funding were available.

Among those interested in developing a scanner, 85% stated they were likely or very likely to use it for diagnostic purposes, and 92.5% reported being very likely to use it for consultations. Furthermore, 100% of participants agreed that guidelines for digital pathology should be established, and 97.4% preferred that such guidelines be developed by the Jordanian Pathology Society (JPS) or the Jordanian Medical Association (JMA).

## Practice of digital pathology and AI

### Equipment access and usage

About 70% of participants reported owning a slide scanner, though all had only one device. Eight used Leica scanners, and three used Motic scanners. These scanners were located in universities, Royal Medical Services (RMS), and the private sector.

Despite this, only 10% of participants reported using digital pathology (DP) in routine diagnosis. Utilization patterns showed that 28% used slide scanners occasionally or frequently, whereas 72% used them rarely. Among current users, 54.8% reported using DP for over half of the cases they signed out.

### Guideline adherence

Among participants using DP, 65% did not follow specific guidelines. Of those who did, 71% adhered to the College of American Pathologists (CAP) guidelines, while 29% followed the Royal College of Pathologists.

### Barriers to adoption

**Figure 3:**
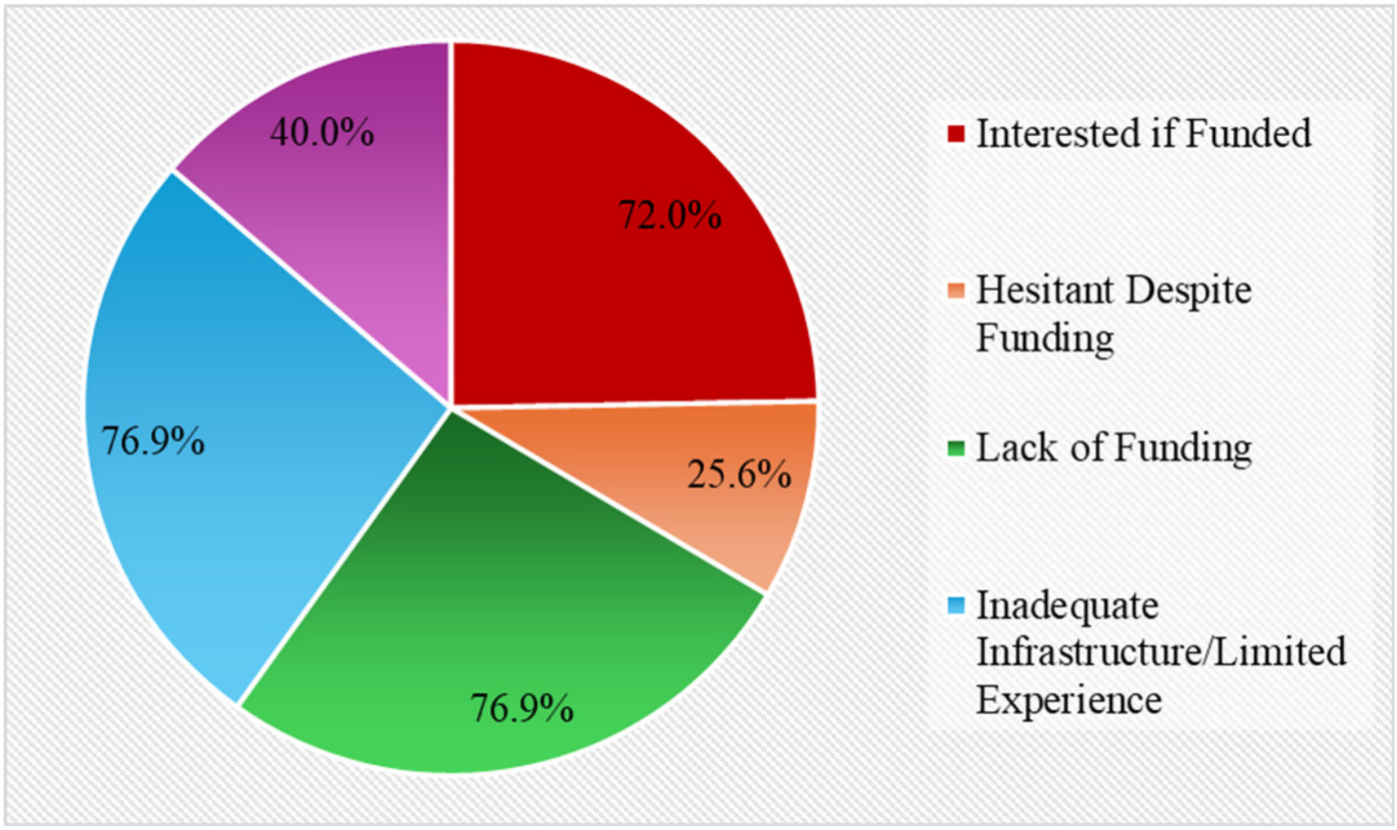
Barriers to the adoption of digital pathology.

The most frequently cited barrier was lack of funding (76.9%), followed by infrastructure limitations and lack of experience. While 72% expressed interest in acquiring DP if funding became available, 25.6% hesitated. Additionally, about 40% cited a lack of interest or a preference for traditional glass slide-based diagnosis Figure 3.

### Applications

When asked about potential uses of digital pathology, participants identified consultation (86.8%), education (73.7%), research (63.2%), primary diagnosis (50%), case archiving (44.7%), and tumor boards (44.7%) as the most relevant. Specifically, 86.8% indicated they would use DP for consultation if available.

### Technical infrastructure

Regarding readiness, 80.6% reported adequate internet speed, and 64.1% confirmed the presence of a pathology Laboratory Information System (LIS). Barcode access was reported by 63%, while 36.8% lacked this system. Though all participants agreed that histology slide quality is essential, only 87.2% confirmed their labs currently meet the required standards.

## Discussion

The findings highlight the diverse institutional affiliations of participants, indicating broad representation across Jordan's governmental, private, and academic healthcare sectors. Private hospitals had the highest representation, followed by universities and the MOH. RMS also had a significant presence. This distribution suggests that the perspectives gathered reflect a well-rounded overview of digital pathology (DP) adoption across various healthcare settings, providing insights into both well-resourced institutions and those facing infrastructural challenges.

A substantial proportion of participants demonstrated moderate to high knowledge of digital pathology, indicating a growing familiarity with technology. However, while access to slide scanners was relatively low, usage patterns were relatively low, with few employing DP for routine diagnosis. This is consistent with global trends, where DP adoption is often hindered by financial and infrastructural limitations despite demonstrated efficiency and diagnostic accuracy benefits (19, 20). Participants with access to scanners predominantly used Leica and Motic models, reflecting a preference for widely recognized brands.

Despite limited current use, there was strong interest in expanding the adoption of digital pathology. Many participants who did not yet have scanners expressed a desire to acquire one, with most indicating they would likely use digital pathology for diagnostic purposes. An even greater number showed a clear intention to use it for consultation, highlighting the potential of telepathology to improve access to expert opinions and support multidisciplinary collaboration (21).

Interest in digital pathology education was markedly high, with 85% expressing a willingness to attend a national congress session. This aligns with global trends where continued education is a key driver for DP adoption (22). AI applications in pathology are gaining momentum, with a notable portion of participants already incorporating AI platforms into their work. Many of these users are primarily utilizing AI for research, while many have also integrated it into computer-aided diagnostics. This growing adoption reflects a broader global shift, as AI-powered tools demonstrate their potential to enhance diagnostic accuracy and reduce workload (8, 23, 24). However, broader adoption of AI in clinical settings remains a challenge due to regulatory concerns and limited expertise (25).

A key observation was that many users of digital pathology did not follow established guidelines; among those who did, most adhered to the standards set by the College of American Pathologists, while others followed the Royal College of Pathologists. There was unanimous support for developing national guidelines, underscoring the pressing need for standardization. Participants also strongly preferred that the Jordanian Pathology Society or the Jordanian Medical Association produce these guidelines. Implementing standardized protocols would help ensure consistency in diagnosis, safeguard data, and promote interoperability among institutions (26).

Financial limitations emerged as the most significant barrier to adopting digital pathology (DP), with many participants identifying a lack of funding as a primary obstacle. In addition to economic constraints, insufficient infrastructure and limited user experience were frequently noted challenges. The hesitancy observed among some participants further suggests that entrenched cultural norms and professional preferences for conventional glass slide diagnosis may also impede integration. Similar patterns have been reported in other contexts, where cost, training deficiencies, and disruptions to established workflows have collectively hindered the broader implementation of DP (27, 28). Overcoming these challenges will require coordinated efforts to secure strategic funding, enhance training programs, and strengthen infrastructure to support sustainable digital transformation in pathology.

The study found that consultation was the most frequently identified intended use of digital pathology, followed by its application in education and research. In contrast, its use for primary diagnosis appeared less common, possibly reflecting ongoing regulatory constraints and workflow integration challenges. Additionally, the reported use of digital pathology for case archiving and tumor board presentations highlights its potential to support multidisciplinary collaboration and enhance long-term data management (3). Most participants indicated that their internet connectivity was adequate to support digital pathology, a critical requirement for efficient digital slide sharing and the effective use of telepathology. However, the availability of Laboratory Information Systems and barcode technology was less consistent across institutions. While high-quality histological slides are fundamental to successfully implementing digital pathology, not all laboratories have confirmed compliance with established quality standards. This highlights the need for strengthened quality control practices to ensure diagnostic accuracy and reproducibility in digital workflows (24).

Although some academic participants indicated that their institutions have incorporated digital pathology (DP) and artificial intelligence (AI) into the pathology curriculum, this remains limited in scope. While such integration represents a positive step, there is a growing consensus that more comprehensive inclusion of DP and AI in medical education is essential to prepare future pathologists for evolving digital demands. As Sriram et al. noted, AI is transforming both learning processes and clinical practice, necessitating curricular reforms. Similarly, Hamilton highlights the shifting landscape of medical education driven by AI, emphasizing the urgency of equipping trainees with the digital competencies required in modern healthcare environments (29, 30).

Our findings reflect regional trends indicating significant interest but limited implementation of digital pathology (DP). A survey of 64 pathologists in National Guard Health Affairs hospitals in Saudi Arabia revealed median acceptance scores of 5.5–6 on the Technology Acceptance Model, indicating overall readiness despite inadequate onsite infrastructure (16). A survey of 127 European and Asian laboratories found that 57% had embraced DP procedures, whereas the rest intended to or rejected them (31). Our study of 40 Jordanian pathologists found that 69.2% have medium or outstanding digital pathology expertise, 85% are interested in its diagnostic use, and 37.5% use AI. This matches regional readiness and worldwide tendencies of limited implementation and strong attitudinal backing. These comparisons show that Jordan follows regional and global trends, but implementation lags behind desire, requiring strategic investment, formal rules, and infrastructure.

It is essential to acknowledge the limitations of this study. The relevance of the results to the broader pathology community may have been constrained by selection bias resulting from the convenience and snowball sampling methods, which may have preferentially included participants with greater interest or experience in digital pathology. The absence of a random sample and a comparative group, such as pathologists from various countries or specializations, further constrains the ability to draw causal inferences or do cross-contextual comparisons. Moreover, the study exclusively employed descriptive statistics, lacking inferential or multivariate methodologies to analyze the relationships among significant variables such as institution type, years of experience, or exposure to AI. Moreover, comprehensive statistical analysis was precluded by the limited sample size. The psychometric properties of the survey remain unassessed, as formal validation metrics such as internal consistency and test-retest reliability were not examined, although it underwent pilot testing for clarity. Future research should consider employing bigger, randomly selected samples, validated instruments, and more advanced statistical methodologies to enhance the rigor and generalizability of findings.

## Conclusion

Despite considerable financial and infrastructural obstacles, this exploratory study indicates a significant interest in digital pathology among Jordanian pathologists. Despite the seemingly elevated claimed knowledge levels, actual performance is somewhat constrained. Mitigating financing limitations, formulating national norms, and integrating DP education into medical curricula may facilitate broader implementation. The growing implementation of AI in research and diagnostics suggests a potentially favorable path for digital pathology in Jordan, contingent upon sufficient resources and expertise. Nonetheless, more investigations with representative samples are essential to validate these findings and guide national efforts.

## Data Availability

The raw data supporting the conclusions of this article will be made available by the authors, without undue reservation.
