# Supplementary material for: Pathologists' user experience in the era of digital pathology: a KAP study in a region of emerging digitization
Source: Front Digit Health. 2025 Sep 1;7:1603985. doi: 10.3389/fdgth.2025.1603985 (PMC12434013; doi:10.3389/fdgth.2025.1603985)
Supplement: Supplementary file 1 [file Datasheet1.pdf]

Supplementary Appendix

Perceptions of Jordanian Pathologists on Digital Pathology (DP)

1. *Mark only one oval.*
Option 1
1. Name

2. Email address

3. Institution

4. On a scale of 1-5, how do you rate your knowledge in digital pathology
Mark only one oval.

	1	2	3	4	5	
No						Expert

5. Do you have a digital slide scanner in your institution
Mark only one oval.
Yes
No

6. If the answer is yes, how many scanners

7. What type of scanner

8. How frequently do you use it on a scale of 1-5
Mark only one oval.

	1	2	3	4	5	
No						Very often

9. If you don't have a digital pathology scanner, are you interested in having one
Mark only one oval.
Yes
No
Maybe

10. If you are given a slide scanner, how likely are you to use it for diagnosis?
Mark only one oval.

	1	2	3	4	5	
						Very likely

11. If you are given a slide scanner, how likely are you to use it for consultation
Mark only one oval.

1 2 3 4 5

Very Likely

12. If you have a digital pathology scanner and you use it, what is the % of cases that are diagnosed using DP *Mark only one oval.*

10%
20%
30%
40%
50%
60%
70%
80%
90%
100%

13. Are you interested in having a session in national congress regarding DP *Mark only one oval.*

Yes
No
Maybe

14. Do you use AI platforms in general (such as ChatGPT)
Mark only one oval.

Yes
No

15. Do you participate in educational activates/sessions dedicated to DP Artificial intelligence (AI)
Mark only one oval.

Yes
No

16. What are the major uses case of AI tools in your pathology lab?
Check all that apply.

Research
Computer aided diagnosis Other:

17. If you have DP, do you follow any guidelines?
Mark only one oval.

Yes
No

18. If yes, what are the guidelines?

19. If you dont have digital pathology or dont follow specific guidelines, do you think we should have guidelines for the use of DP? *Mark only one oval.*

Yes

No

20. Do you think that regulatory agencies/ gov / JFDA should produce regulatory guidelines? *Mark only one oval.*

Yes

No

21. Would you prefer Jordan pathologist society/ JMA come up with those guidelines? *Mark only one oval.*

Yes

No

22. If funds are available for DP, would you be interested in acquiring it and using it for diagnosis? *Mark only one oval.*

Yes

No

Maybe

23. What are the challenges that prevent the adoption of DP in your opinion?

Check all that apply.

Lack of funds

Lack of infrastructure

Lack of experience

Lack of interest

Prefer the old fashioned way Other:

____

24. Do you think you have adequate internet speed to use DP if available? *Mark only one oval.*

Yes

No

25. If you have digital pathology, what would be the primary uses?

Check all that apply.

Research

Primary diagnosis

Archiving cases

Education

Tumor boards Consultation cases

Other:

____

26. Do you have pathology LIS (laboratory information system) in pathology laboratories

Mark only one oval.

Yes

No

27. What is the availability of barcoding in your lab from 1-5

Mark only one oval.

	1	2	3	4	5	
						Always available

28. Do you think the quality of histology slides are important for satisfactory DP

Mark only one oval.

Yes

No

29. Do you think your lab produces histopathology glass slides that meet the quality standards required for digital pathology scanning ("DP ready" slides)?

Mark only one oval.

Yes

No

30. If you work in a university setting, does the curriculum contain lectures on DP/AI

Mark only one oval.

Yes

No

I don't work in a university setting

31. How would you rate this survey out of 5

Mark only one oval.

1	2	3	4	5
